# Face detection based on a human attention guided multi-scale model

**DOI:** 10.1007/s00422-023-00978-5

**Published:** 2023-12-01

**Authors:** Marinella Cadoni, Andrea Lagorio, Enrico Grosso

**Affiliations:** Dipartimento di Scienze Biomediche, Università di Sassari, Viale San Pietro 43B, 07100 Sassari, Italy

**Keywords:** Multiscale face model, Facial visual attention, Attention-guided model, Multiscale face detection

## Abstract

Multiscale models are among the cutting-edge technologies used for face detection and recognition. An example is Deformable part-based models (DPMs), which encode a face as a multiplicity of local areas (parts) at different resolution scales and their hierarchical and spatial relationship. Although these models have proven successful and incredibly efficient in practical applications, the mutual position and spatial resolution of the parts involved are arbitrarily defined by a human specialist and the final choice of the optimal scales and parts is based on heuristics. This work seeks to understand whether a multi-scale model can take inspiration from human fixations to select specific areas and spatial scales. In more detail, it shows that a multi-scale pyramid representation can be adopted to extract interesting points, and that human attention can be used to select the points at the scales that lead to the best face detection performance. Human fixations can therefore provide a valid methodological basis on which to build a multiscale model, by selecting the spatial scales and areas of interest that are most relevant to humans.

## Introduction

Face detection is a fundamental preliminary step of all facial analysis algorithms, from face tracking to identity verification, mood recognition, and many more, which are applied to countless consumer applications and devices characterized by intelligent, vision-based, human–computer interaction.

According to the categorization proposed by Zafeiriou et al. [1], face detection techniques can be broadly divided into two main classes: the family of algorithms based on iconic rigid templates and the family of algorithms represented by Deformable Parts-based Models (DPM).

The first family of algorithms, that we better denote as holistic methods, comprises old handcrafted template-based methods [2] and appearance-based methods, where the models are learned from a set of training images which should capture the representative variability of the face [3]. Among the family of rigid templates are a series of improvements based on boosting or statistical classification techniques; among these, the Viola–Jones architecture [4] has been a source of inspiration for many variants such as SURF cascades [5] or the aggregate channel features method for multi-view face detection [6]. The rigid template category finally includes algorithms based on deep-learning architectures such as convolutional neural networks (CNNs) and Deep CNN (DCNNs) [7, 8] that have been successfully introduced for face detection tasks. In the last few years, we have witnessed the advance of such kind of detectors. Real-time face detection based on YOLO [9, 10] has outperformed, with stronger robustness and faster detection speed, all previous detection methods [11, 12], and it has recently been surpassed by the pixel-wise face localization RetinaFace [13] which, notably, makes use of manual face annotations, and by the Multi-task Cascaded Convolutional Networks (MTCNN), which inputs a scale pyramid of the original face image into a three stage framework that progressively refines the bounding boxes found initially [14].

Deformable parts-based models [15], on the other side, see the human face as a collection of parts trained side by side with the face using a spring-like constraint. Very often DPMs are multi-scale models [16], as they bring together holistic (at a coarse scale) and local (at fine scale) information. Support vector machines (SVM) are typically used to find the parts and their relationships; furthermore these parts are adjusted to operate efficiently with the HOG descriptors [17]. As shown by Zhu and Ramanan [18] and Mathias et al. [19], these models can give impressive results even though they are trained with a few hundreds of faces, which is an important advantage over neural networks that usually require billions of examples. The downside is that DPMs follow a top-down, multi-scale approach based on handcrafted features that is arbitrarily defined by a human specialist and then validated on the basis of heuristics. On the contrary, deep learning-based models are bottom-up, where interesting regions of the image emerge through internal layers [20], but they are not intrinsically scale-invariant, an ability that humans can achieve after a single exposure to a novel object [21], and lack explainability (human understanding of what actually happens within the network is so far very limited). Deep learning-based models in general seem to show moderate correlation with human visual attention [22], but the higher the correlation, the better they perform at classification tasks [23], which is one of the reasons why it is desirable to build machine models that incorporate human attention.

The humans visual system seems to be wired to detect faces. This ability has been recently explored in Baek et al. [24] where, by using a hierarchical deep neural network model of the ventral visual stream, it was found that face selectivity arises without any prior training. While studies on the performance of the detection step of face perception are scarse, the results in [25] seem to point to the fact the human visual system is very efficient at detecting faces, and that failures in face processing tasks such as identification arise after the segmentation step. An interesting study [26] that investigated the detection performance by observers on images that were falsely detected as faces by the Viola–Jones algorithm [4], found that human observers are more prone to classify these as faces compared to images of non-faces. However, humans where able to correctly classify about $20\%$ of these images as non-faces, and in a short time of 20 ms. Despite the fact that there are not direct comparisons between humans and algorithms on face detection, from the existing literature it seems that humans can compete and likely surpass face detection algorithms if given enough time to observe the images.

To overcome the dichotomy between top-down algorithmic models and bottom-up black-box models, in this paper we apply the well-known scale-space theory developed by Lindeberg [27] to construct a multi-scale model that derives from a pyramidal bottom-up process. More in detail, we show that persistent points that characterize some kind of visual information like a face easily emerge at different scale levels; as a consequence, it is not necessary to pre-determine the number of features or the spatial scales that better represent the image information. Considering the extreme complexity of such a pyramidal model, the choice of essential scales, i.e. the scales that contain most of the image information, becomes a crucial problem. Here we explore if human visual attention, specifically the fixations of humans on face images, can guide the selection of the essential scales in building the proposed face model.

In [28] the authors find that although different human observers adopt different strategies when looking at the same face, when cumulating the fixations of all observers the attention areas tend to cluster around some facial features (e.g., the areas around the eyes, the nose and the mouth). Also, several authors argue that humans gaze follow a coarse to fine pattern when observing natural scenes [29].

To see if human visual attention could be embedded in our face model, we specifically acquired the gaze paths of 20 observers on a face images subset of the Karolinska dataset [30]. By performing a cluster analysis on the features extracted using the scale-space theory and the fixations we showed that the selection of the spatial scales can be guided by human attention. In particular, we found that by using only two spatial scales defined by human attention the model performs better or similarly than it does when using other available scales. Attentional mechanisms typical of humans can thus drastically simplify the construction and the use of a multi-scale model.

The paper is structured as follows: In Sect. 2 the bottom-up multiscale model is introduced; in particular the construction of a 10-scales pyramid, from the Labeled Faces in the Wild (LFW) dataset, and the training of the HOG model descriptors at different scales is detailed. The collection of a face-based fixation dataset and the clusterization leading to the selection of the most representative spatial scales is described in Sect. 3. Section 4 presents some extensive results of the experimental application of the model to a large database and gives an the comparison with other state-of-the-art face-detectors. Finally, the conclusions are drown in Sect. 7.

## Bottom-up multi-scale model

### Image features extraction

The extraction of the features used in the proposed model is based on the scale-space theory developed by Lindeberg [27]. Given an image $f:\mathbb {R}^2\rightarrow \mathbb {R}$, a Gaussian scale-space representation is defined to be a map:

1 $$\begin{aligned} L(x,y;\sigma )=\int _{(u,v)\in \mathbb {R}^2} f(x-u,y-v) \,g(u,v;\sigma )\, \text {d}u\, \text {d}v\nonumber \\ \end{aligned}$$

where $g(u,v;\sigma )=\frac{1}{2\pi t}\text {e}^{-(u^2+y^2)/2\sigma }$ is the Gaussian kernel of variance $\sigma$, which represents the scale parameter. Starting from this representation, given a scale $\sigma$, Lindeberg defines four spacial differential operators based on the Hessian matrix $H_L$ of *L*, each leading to a particular type of feature points. Among the operators we chose the Laplacian, defined as:

2 $$\begin{aligned} \nabla ^2 L = L_{xx}^2+L_{yy}^2 = \lambda _1+\lambda _2, \end{aligned}$$

where $\lambda _1, \,\lambda _2$ are the eigenvalues of the Hessian matrix, or the principal curvatures of $L(,,\sigma )$.

Extrema of $\nabla ^2 L$ correspond to dark or bright blobs, according to whether the Hessian is positive or negative definite. Edges will also be detected, but they are discarded to improve the repeatability of points detection [27].

For the Laplacian operator a pyramid of 10 layers was built, one for each scale $\sigma _k = \sqrt{n}, \textrm{for}\, n=2^{(k-1)}, k=1, \ldots , 10$, starting from the original image, and halving the image every two steps. For each scale, local extrema were calculated with respect to the image coordinates. Most of these extrema are likely to persist across two or more scales. Scale linking as described in Lindeberg [27] has been carried out to select their strongest response across scales.

### Model construction

To learn a model of the human face, we selected images of faces from the LFW dataset [31]. In particular we considered the subset of LFW [32] consisting of one or more aligned images of 5749 different subjects. The images are not controlled for illumination, backgrounds, slight face poses, expressions or the presence of accessories such as glasses, hats etc. We selected a subset $T_N$ by randomly picking *N* identities from the training set of LFW and choosing the first image from their folder. For each of the *N* images in $T_N$ we build the 10 layers pyramid, applied the operator $\nabla ^2 L(x,y,\sigma )$ at each scale and then performed scale linking to extract the extrema in scale-space.

For each image $I_i\in T_N$ we get 10 sets of features $F_{\sigma _{k}}(I_i)$, one for each scale. At this stage, for each scale $\sigma _k$, we considered the union of all the feature points sets $U_{F_{\sigma _k}}(N) = \bigcup _{i=1,\ldots ,N}F_{\sigma _{k}}(I_i)$. For a sufficient number of images (we started from $N=25$ in our experiments), $U_{F_{\sigma _k}}(N)$ can be thought as a set of random samples drown from a distribution whose probability density function can be estimated via a kernel density estimation (KDE). The resulting probability density function $\text {PDF}(\nabla ^2 L,\sigma _k)$ will be our feature density map at the scale $\sigma _k$.

**Fig. 1:**
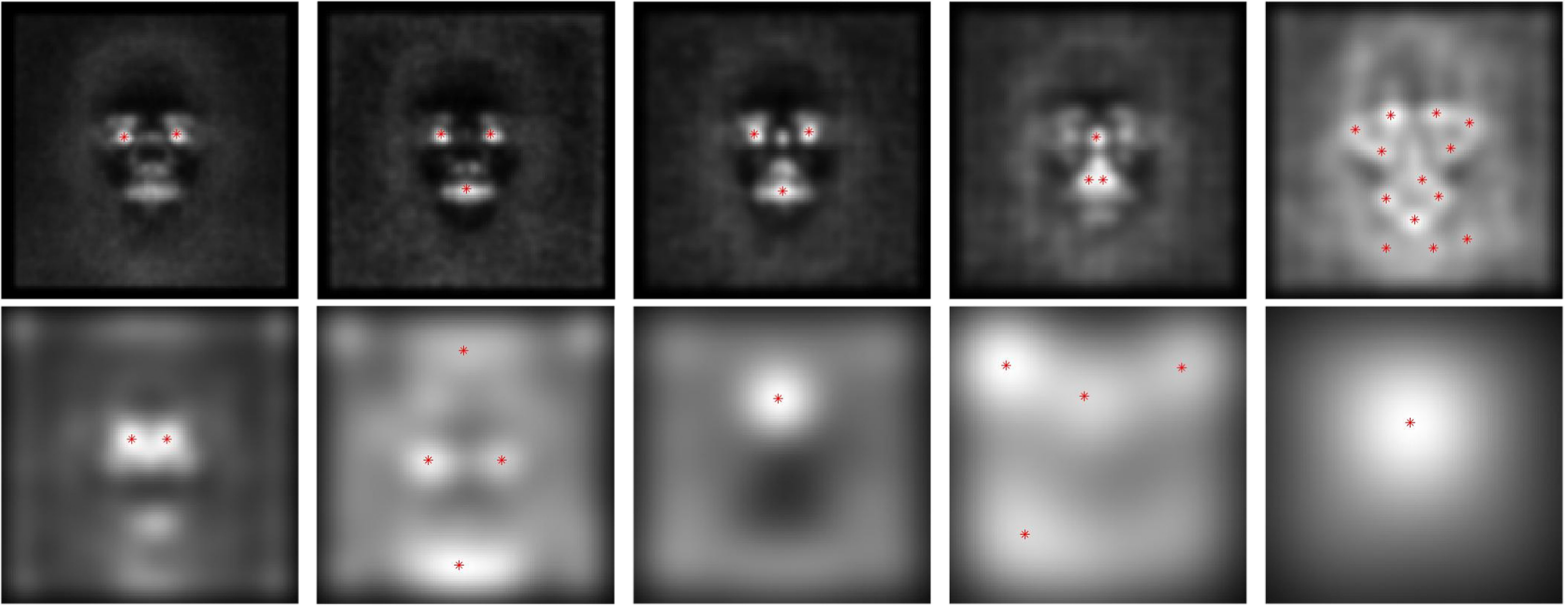
Projections of the density maps on the image plane and strongest local maxima for different spatial scales (top left image corresponds to $\sigma _1 = 1$, bottom right to $\sigma _{10} = 16 \sqrt{2}$)

In Fig. 1 we can see the projections of the density maps $\text {PDF}(\nabla ^2 L,\sigma _k)$ on the image plane, one for each scale for $k=1,\dots , 10$. The brighter the area the higher the value of the probability density function. The density maps were obtained by applying the scale-space extrema extraction to $N=2000$ randomly picked images. We can clearly see different face patterns emerging: at the smaller scales, the Laplacian generates a feature map where the strongest areas are around the eyes, the nose and the mouth, while the contour of the face oval is weakly outlined. As we go up the scale pyramid, we can see other interesting patterns, such as the area at the top of the nose and in between the eyes. At the top of the pyramid, the Laplacian responds the oval of the face. This result suggests that by cumulating the features at each scale a representation of the face naturally emerges, which strengthens the hypothesis in Baek et al. [24] where, in a very similar way, the face representation emerges without any supervised training in the deep neural network model of the ventral visual stream.

**Fig. 2:**
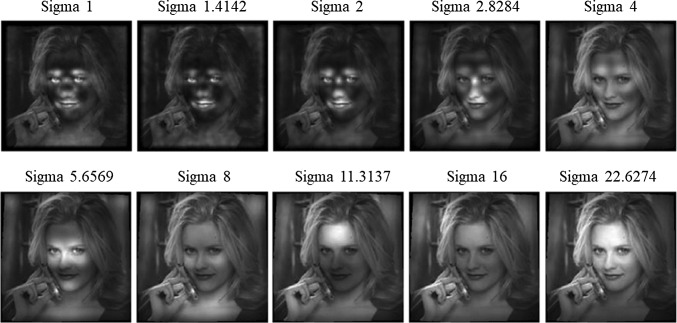
Feature density maps for the operator $\nabla ^2 L$ for the 10 scales projected onto a face image

By projecting the feature maps on top of one of the face images used to generate them, as in Fig. 2, we can clearly see what areas of the face are learned by the model.

### Feature maps convergence

**Fig. 3:**
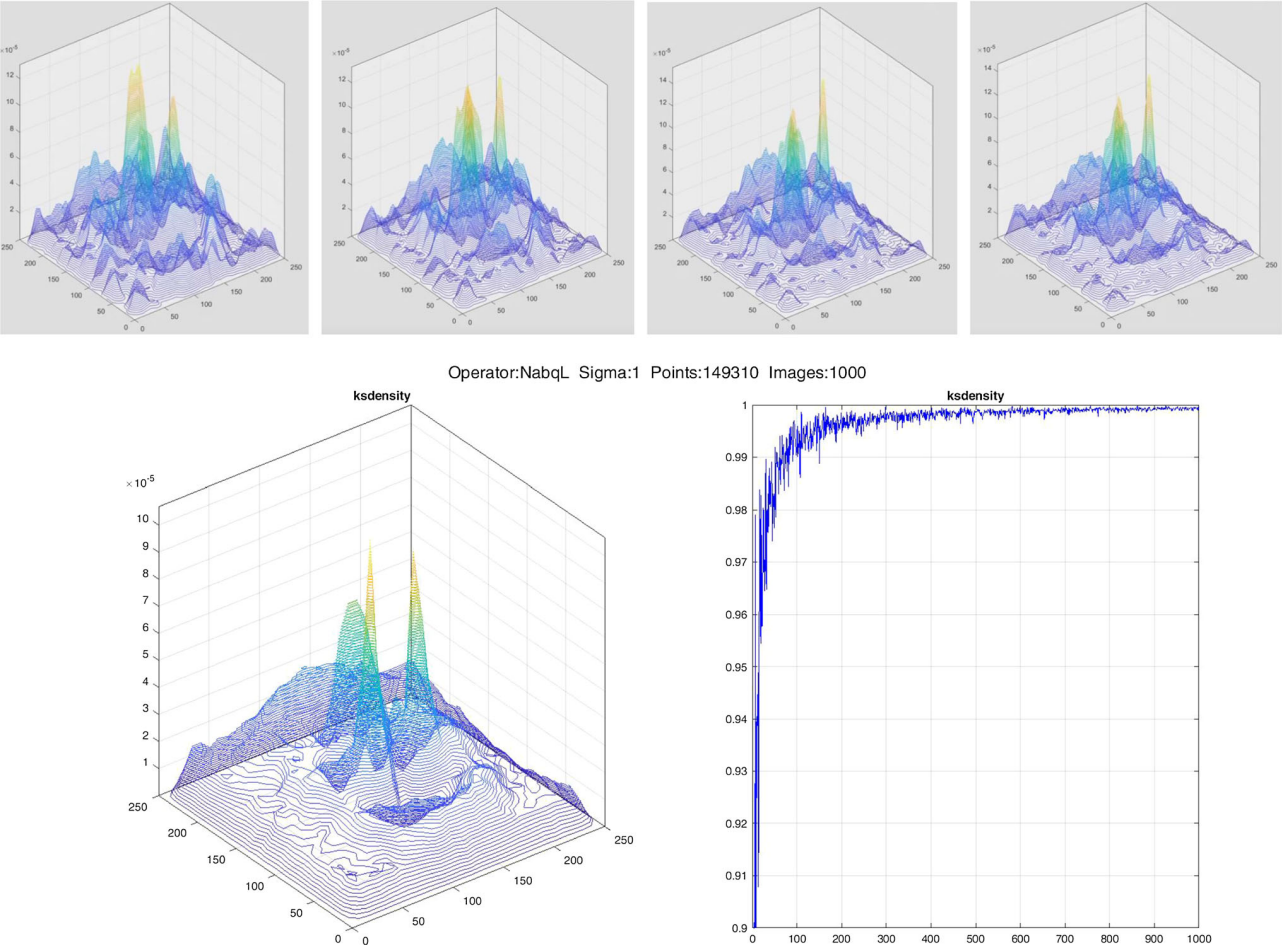
Top row: feature maps deriving from points extracted at scale $\sigma _{2} = \sqrt{2}$ from $25,\, 50,\, 75$ and 100 face images. Bottom row, left: density obtained with 1000 images. Bottom row, right: graph of number of images on the horizontal axis and Bray-Curtis similarity on the vertical axis

In the previous sections, we have defined the feature maps as the probability density functions estimated by KDE on *N* face images. Here we see how they change as *N* increases. To evaluate it, for each scale we generated the feature maps starting with the distribution obtained from features extracted from 25 random images and updating it with the features extracted from a newly added image. Each time we estimated the density via KDE and measured the difference between one density map and the next using two different similarity indexes, the Bray–Curtis index and $1-\text {JSD}$, where JSD is the Jensen–Shannon divergence. The two similarity indexes were consistent. We can say that the probability density functions come from the same distribution if the two indexes are equal to 1. In Fig. 3, the graph on the bottom right shows the similarity scores (vertical axis) as the number of images *N* increases (horizontal axis). We can see that after a rough start, for $N>200$ the curve is very smooth with index values always above 0.99. In fact, as few as 100 images could be used to generate the density maps, but since the database we choose is not controlled for poses, expressions and background we might want to use 1000 or 2000 images to reach a good symmetry of the face areas.

The model here proposed is generated directly from the pyramid of feature density maps. More in detail, the process analyzes all levels of the pyramid and for each determines where the relevant information is located. For a scale $\sigma _k$, we find the global maximum $\max \{\text {PDF}(\nabla ^2 L,\sigma _k)\}$ of the feature density map $\text {PDF}(\nabla ^2 L,\sigma _k)$. We then find all local maxima and we select those whose value is not less than $70\%$ of the global maximum. Figure 1 shows an example of the final result. Notably, the number of significant points is not excessive and it is well distributed over the full scale range.

### HOGs extraction and training

**Fig. 4:**
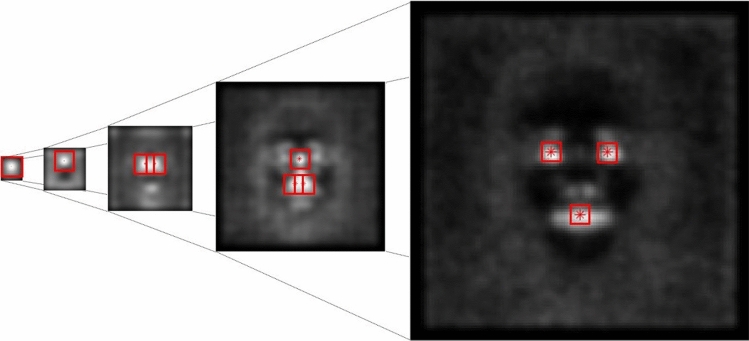
Example of bottom-up multi-scale pyramid. At the top of the pyramid (first image on the left), the patch includes the oval of the face on the image at scale $\sigma _{10}$. The next scale level selected is $\sigma _8$, and the patch is over the forehead of the face. By keeping going down the pyramid every other scale the patches will cover smaller and smaller areas of the image, capturing the area around the pupils at the last layer of the pyramid

Having extracted the most salient points from each scale, we can now use this information to train a classifier. We use one image from each of the first 3000 subjects of the LFW dataset. After extracting the maxima of the Laplacian at the scales corresponding to $k=1,\dots ,10$ as described in section 2, we consider squared patches of size $25\times 25$ pixels, centered at the density maxima and having identical size at each pyramid level. Figure 4 shows a general example of the pyramid with the patches centered at the maxima of the $\nabla ^2 L$ operator. From each patch, 9 HOGs are extracted so that the resulting vector has 144 components. Note that, for practical implementation reasons, in the experiments we only consider scales corresponding to $k=1,\dots ,7$; we could have started from the scale $\sigma _{10}$ but this would have required a smaller patch at the expense of a less descriptive HOG vector. The vectors are used to train a SVM classifier with the first 1000 positive sample images from the LFW dataset and the first 1000 negative sample images from the val2017 partition of the COCO2017 dataset [33], which consist of a random selection of images.

**Fig. 5:**
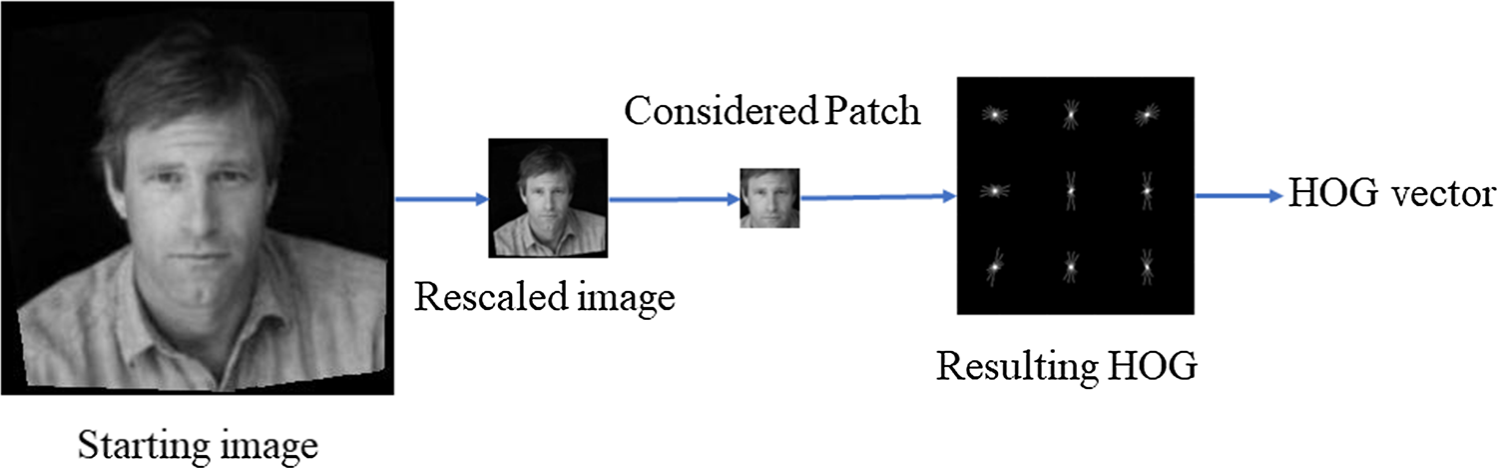
Example of HOG extraction

## Fixations guided scales selection

The number and proximity of scales in Lindeberg [27] is chosen in order to obtain a robust set of extrema in scale space. However, adjacent scales will often generate very similar features, and there are not, a priori, meaningful criteria for selecting one scale over another and so to decide what the most relevant scales to build the model are. To guide the scale decision process, we propose to use the human fixations from the dataset Uniss-FFD [34]. The fixations were collected with a Tobii Eye Tracker [35] from 20 observers (10 males and 10 females), 18 of which were university students 20 to 24 years of age, while 2 were academic staff aged 30 and 50. The observers were shown a selection of images of the first 20 male and the first 20 female individuals from the KDEF dataset [36], namely the front-facing images of the expressions “neutral”, “happy”, and “sad”, resulting in a total of 120 images which were randomly arranged in a sequence. The images in the sequence were free-viewed by the participants, each image was shown on the screen for three seconds and interleaved with two seconds of black screen. The maximum number of fixations for any one face by any observer was 14, but there were very few of them, so, for our purposes, we considered the first 12 fixations. The three seconds viewing time was chosen to provide enough time for the exploration of the face, which some recent works argue can be split into three phases: A first saccade is followed by an initial exploration characterized by a gradual broadening of the fixation density which then reaches a steady state after about 10 fixations [37].

Confirming previous findings (see for instance [28]), by analyzing the Uniss-FFD dataset it turns out that each observer has her/his own strategy to look at faces. Whether this is holistic or analytic, the observations areas tend to cluster around some facial features such as the eyes, the central part of the face around the nose (often shifted on one of the sides) or the mouth area.

**Fig. 6:**
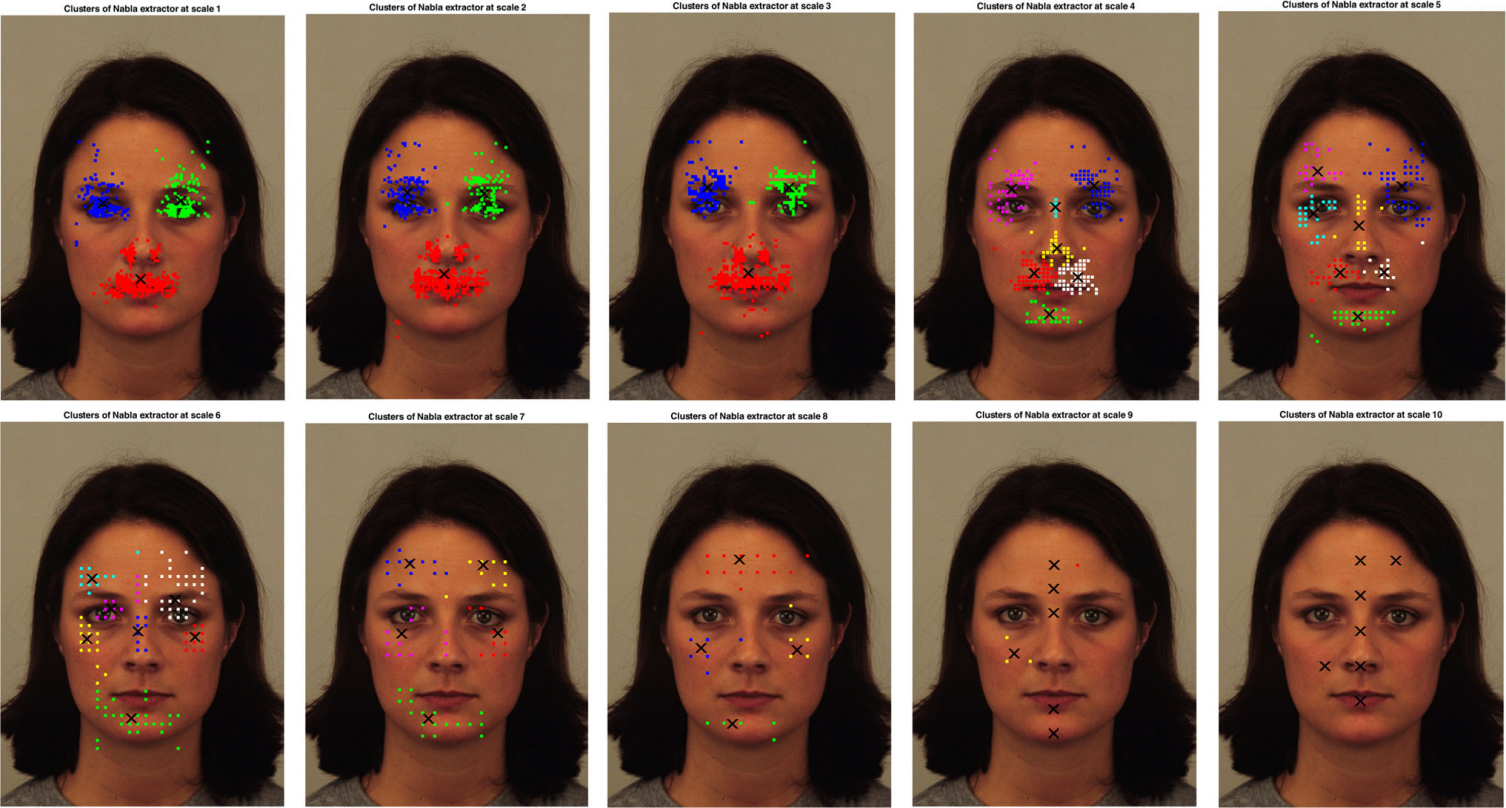
Clusters of nabla maximum points at the 10 scales

**Fig. 7:**
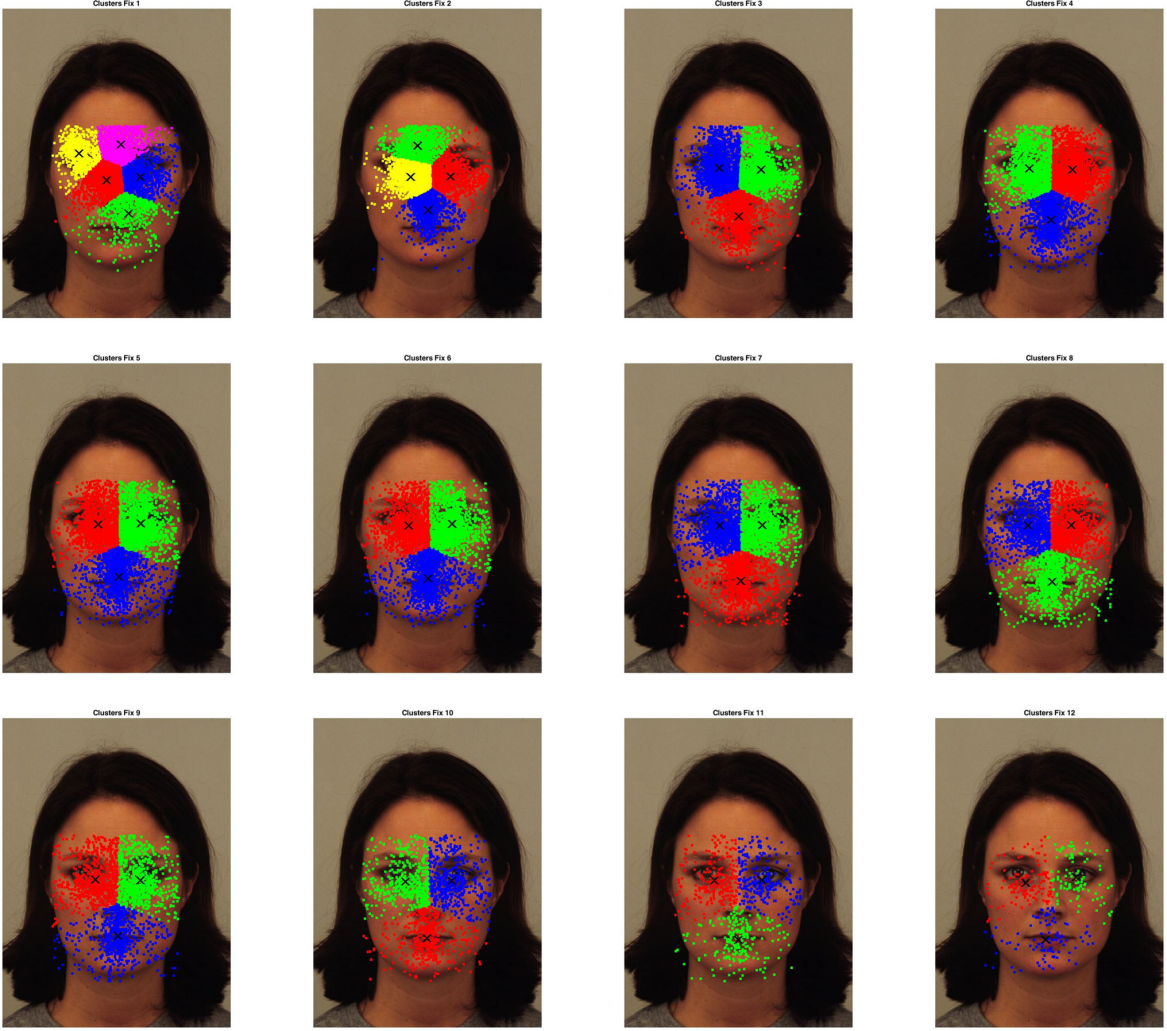
Clusters of cumulative fixations from all observers over all images, segmented by number of fixation

**Fig. 8:**
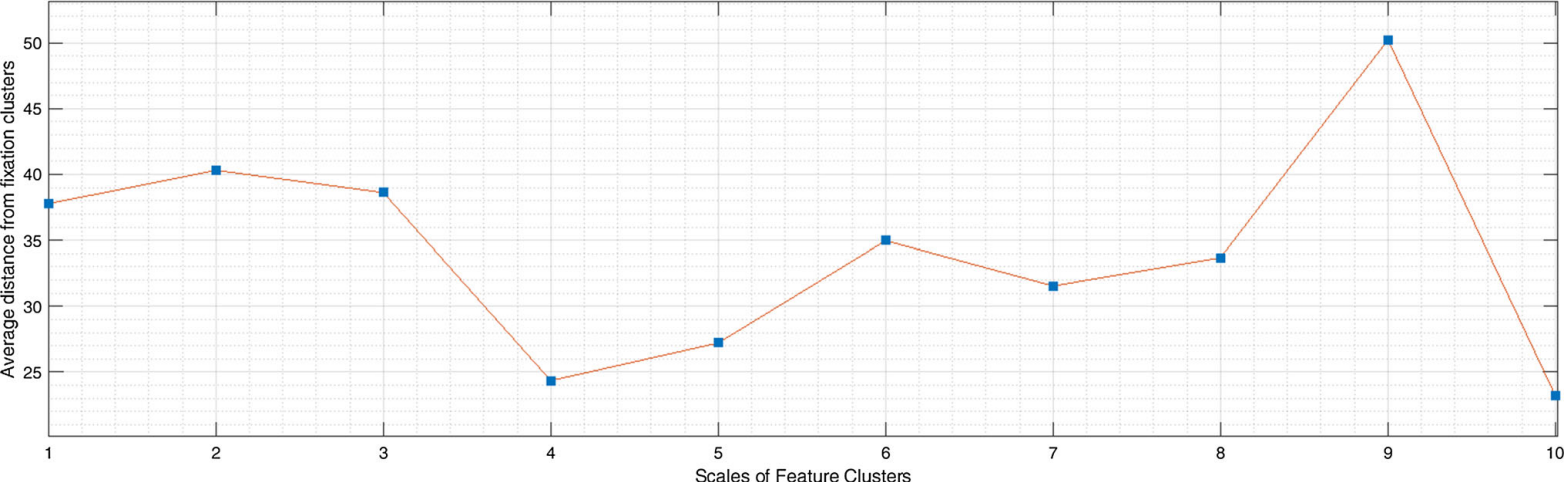
Plot of average distance of features clusters from the fixation clusters, as a function of features scale

To compare the features at the various scales to the human fixations, a cluster analysis of all sets of features and fixations is performed. Some authors argue that when observing scenes, the eye movement follows a scanning strategy that starts on a coarse scale and then progressively focuses on finer scales. Whether this interpretation is correct or not, we can see in [29] that the pattern of fixations and saccades when observing scenes consists of a rapid increase of fixations duration for the first three fixations, followed by a further slight increasing duration for later fixations, while the amplitude of the first three saccades also rapidly grows but it reaches a maximum at around fixation 5 and then gradually decreases. This pattern suggests a quick, ample scan at the start of the observation and a more focused, local scanning strategy from fixation 5 onwards. Since the features were extracted from the coarser to the finest scales, to include the “coarse to fine” pattern of fixations we choose to compare all scales to all fixations (from the first to the 12th). The fixations of all observers were therefore partitioned into 12 sets $F_i, \, i=1,\dots \,12$, where each $F_i$ contains the i-th fixations of all observers on all face images, while the features of $\nabla ^2 L$ were extracted and are collected into the sets $\nabla ^2 L_k$ for $k=1,\ldots ,7$. Each of these sets is clustered using a k-means algorithm with 10 repeated initializations and the optimal number of clusters in the integer interval [1, 7] is chosen using the Davies–Bouldin index, which performs well in case of overlapping clusters. Let $C(\nabla ^2 L_k)$ denote the set of cluster centers found for the set of features $\nabla ^2 L_k$ at scale *k*, and $C(F_i)$ the cluster centers for the i-th fixations set $F_i$. If $|C(\nabla ^2 L_k)|=n_k$, for each cluster center $c_{k_j}\in C(\nabla ^2 L_k), \,j=1,\ldots , n_k$, the closest cluster center in $C(F_i)$ is found, and the distances between these centers are averaged to get $d_{k,i}$. We repeat the search for the minimum distance cluster centers for each $i=1,\dots ,12$ so, for each scale *k*, we get the vector $D_k(\nabla ^2\,L) =\{d_{k,1},\ldots ,d_{k,12}\}$. By averaging it, we get the distance $d_{k}$ relative to the comparison of the features $\nabla ^2 L$ at scale *k* with all the human fixations $F_i$. The procedure to find the distances between the cluster centers of the features and the cluster centers of the fixation was repeated 100 times to avoid instability of the k-means initialization. Figure 6 shows the Laplacian features clusters at scales $k=1,\dots ,10$, while Fig. 7 depicts the clusters for the human fixations. Figure 8 shows the plot of the average distances vector. We select the scales for which the distance vector has a local minimum, which correspond to scales where features clusters are more similar to fixations clusters. From the graph, this would mean selecting the scales $\sigma _4$ and $\sigma _7$ and $\sigma _{10}$, although the model will start with scale $\sigma _{7}$ as $\sigma _{10}$ proved too small for the descriptor to be meaningful.

## Multi-scale model applied to face detection

The proposed trained model can be promptly used to detect faces in images. In particular, starting from the coarsest scale of the model ($\sigma _7$ in our case), the search gradually proceeds to finer scales that may or may not confirm the presence of significant details.

Given an image, since a priori we do not know what the sizes of the (possibly contained) faces are, the image is first resized to match the size of the coarsest scale of the model ($32$ pixels). Then, for each pixel of the image, a patch of size $25\times 25$ centered at the pixel is evaluated by extracting the HOGs, which are then fed into the SVM classifier. If the result is negative, the pixel is discarded, otherwise it is analyzed at the successive (finer) scale of the model (e.g., $\sigma _6$ if all scales were used; $\sigma _4$ if the model guided by human fixations were used). If all scales of the model give positive results, a face is assumed to be present in the image with a dimension given by the coarsest scale. If not, the original image is resized to match the size of the successive (finer) scale of the model and the multi-scale analysis just described is repeated. The process stops as soon as all the scales foreseen by the model cannot find a corresponding level of analysis on the original image (i.e. when the dimension of the possibly contained face in the original image is too small to be processed at any of the scale levels of the model).

## Experimental setup

To evaluate the face detection capability of the proposed model we run extensive experiments on the Face Detection Data Set and Benchmark (FDDB) [38], which contains the annotations for 5171 faces in a set of 2845 images divided into 10 folders. The images were taken from the Faces in the Wild data set. Annotations were carried out by observers, who were instructed to not annotate as faces image regions containing faces that where rotated more than $90^{\circ }$ from the camera, or if neither of the two eyes (or glasses) were visible. They were also requested to reject a face region if they were unable to estimate its position, size, or orientation. A region was finally labeled as a face based on aggregating statistics of the labeling from multiple observers.

We designed several test protocols to evaluate the segmentation performance of the face model with and without human attention guidance. With the proposed method, the confidence score of each detected image region can only assume two values (0 and 1), as we are using a binary classifier. For each input image, the output is a (possibly empty) set of image regions of rectangular shape classified as faces. The size of the detected rectangles is related to the coarsest scale at which the face was found.

To see which scales combinations had the best segmentation capability we run a preliminary test on a subset of FDDB. Since each of the 10 folders of FDDB contains random images from Faces in the Wild, we choose the first folder as a representative subset of the whole dataset and we run experiments by considering face models with the most meaningful scales combinations, excluding adjacent scales that have similar Laplacian extrema and ultimately similar information:

1. $\text {FM}_{\{i\}}$, with $i\in \{1,\ldots ,7\}$ are the models based on a single scale $\sigma _{i}, \, i=1,\ldots , 7$.
2. $\text {FM}_{\{5,3\}}$ and $\text {FM}_\{5,1\}$ are the models based on two scales, with initial scale $\sigma _5$.
3. $\text {FM}_{\{7,i\}}$, with $i \in \{5,4,3,2,1\}$ are the models with first scale equal to ($\sigma _7$). This set includes the model $\text {FM}_{\{FG\}}$ based on the scales that are most similar to human fixations, namely $\sigma _7$ and $\sigma _4$.
4. $\text {FM}_{\{7,5,2\}}$ and $\text {FM}_{\{7,4,1\}}$ are the models based on three scales with initial scale $\sigma _7$.
5. $\text {FM}_{\{7,6,5,4,3,2,1\}}$ is the models based on all scales.

**Fig. 9:**
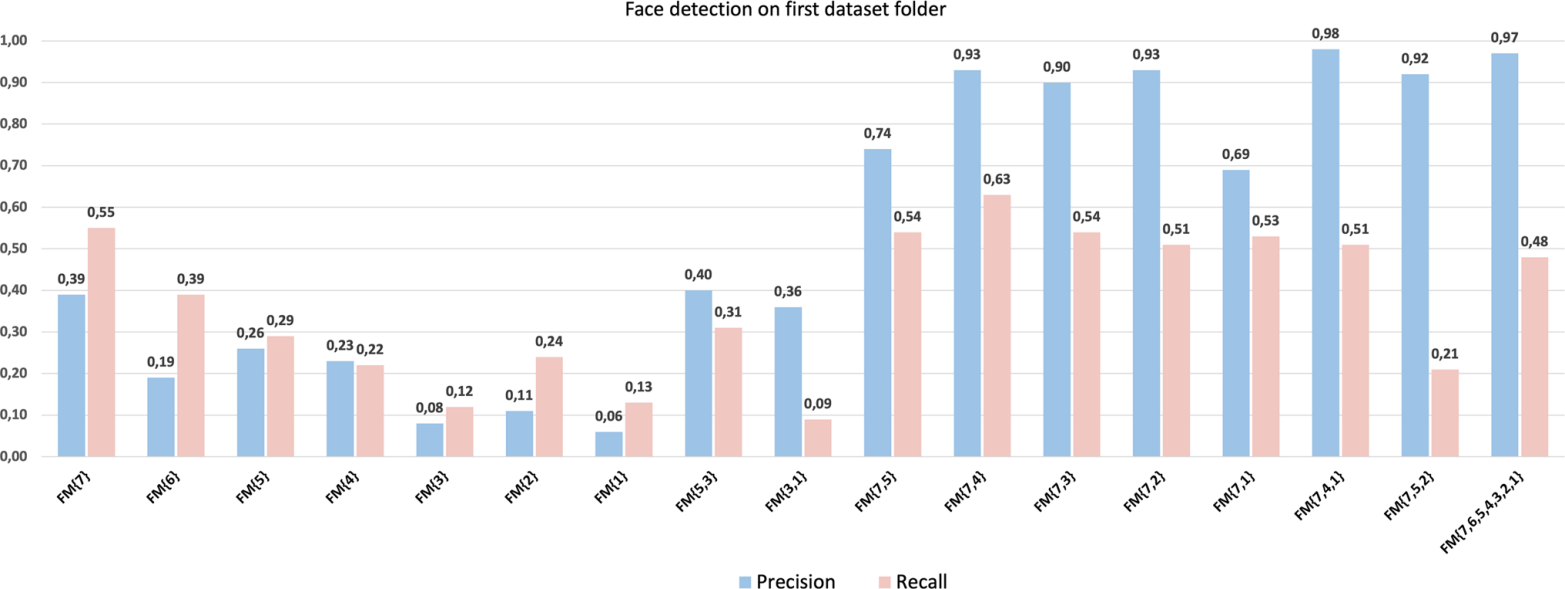
Precision and Recall of various Face Models for face detection on the first folder of the FDDB database

The face detection performance of these models is reported in Fig. 9. As expected, a single scale is not enough for the model to perform face detection. The human-driven model $\text {FM}_\{\text {FG}\}=\text {FM}_\{7,4\}$ proves to be the most accurate if we consider both precision and recall, while the models that could challenge it are, starting from the left-hand side of the bar plot, the tenth and the last six. All these models are based on two or more scales that start at $\sigma _7$ and they are the ones we included in the experiments on the whole FDDB.

For the full experiments, all images contained in all the 10 folders of FDDB were processed to search for faces. No training was performed on FDDB. To establish if the fixation guided model could compete with the most promising models in the experiments restricted to the first folder, we run all the experiments listed in points 4 and 5 above.

## Experimental results

As prescribed in [38], the measure we use to evaluate the regions selected by a face detection model is the discontinuous Intersection over Union (IoU), and the suggested threshold to establish a true positive region is set to 0.5. It is then suggested that if you have a classifier that outputs a confidence score, the ROC curve should be calculated as the confidence score varies. In our case we only have two possible confidence scores (0 and 1), so to establish the performance of the fixation guided model with respect to models based on other scales we evaluate the precision and recall at the IoU suggested threshold 0.5 as well as at different values of IoU in the range [0, 1].

**Fig. 10:**
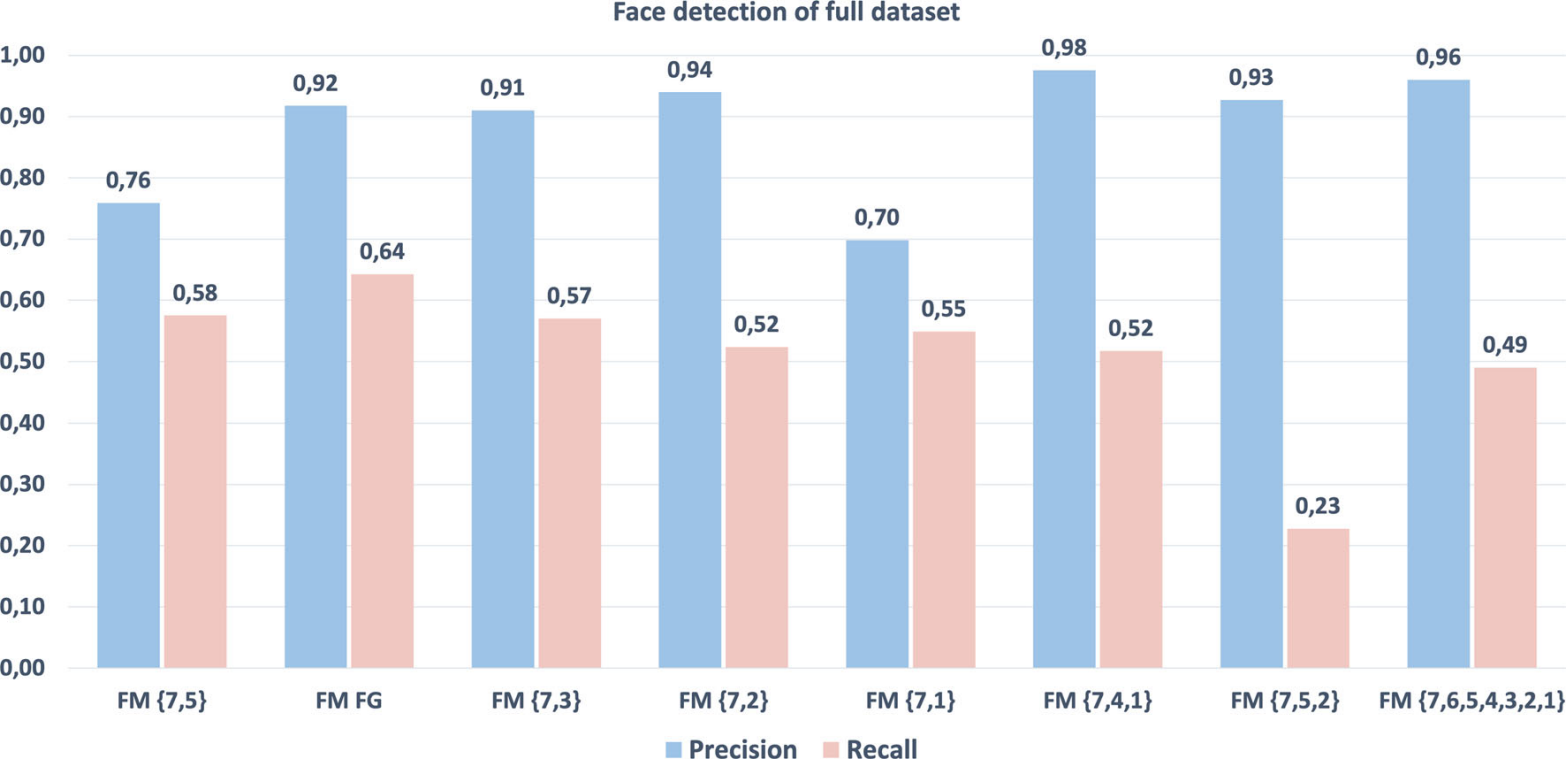
Precision and recall at $\text {IoU}=0.5$ for $\text {FM}_{\text {FG}}$ and the competing face models based on other scale combinations

The results of the face detection are shown in Fig. 10. The $\text {FM}_{\text {FG}}$ has the best scores among all models based on two scales $\text {FM}_{\{7,i\}}, i=1,2,3,5$. The models $\text {FM}_{\{7,4,1\}}$, $\text {FM}_{\{7,5,2\}}$ and $\text {FM}_{\{7,6,5,4,3,2,1\}}$ provide the highest precision at the expense of a lower or much lower recall, which can be explained by the fact that more genuine faces are discarded when adding one scale as the regions might be discarded by the classifier at that scale. The human-based model allows an overall better precision and recall of all of the other models, at the same time limiting the processing to only two scales and so decreasing the computational burden.

**Fig. 11:**
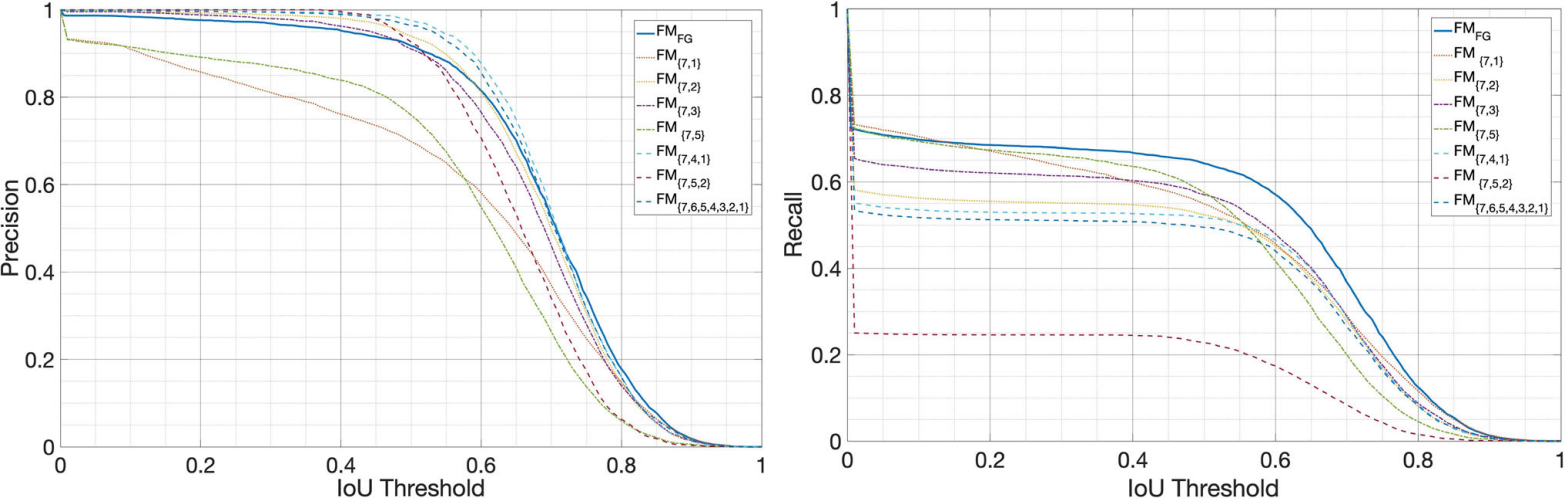
Precision and Recall at varying IoU thresholds for $\text {FM}_{\text {FG}}$ and other face models based on various scale combinations

In Fig. 11 we can see the precision (left) and the recall (right) as IoU varies. $\text {FM}_{\text {FG}}$ has a high precision in the interval of $\text {IoU}=[0,5]$, comparable to the models based on three scales, and a much higher recall than the three scales or the all scales models. Considering both precision and recall, we can conclude that the model guided by human fixations is the one with the best performance, so this first result tells us that human attention can successfully guide the selection of the scales.

As an example, in Table 1 we can see an image from the dataset FDDB and the results of the face detection of the models based on different scales. The $\text {FM}_{\text {FG}}$ detects all faces while all the others miss one or more.

**Table 1:**
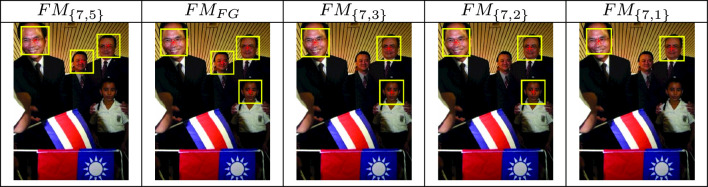
Examples of face detection using our face model at different scales

To compare our human attention guided model with the state of the art in face detection we see how precision and recall vary as the IoU threshold varies in the range [0, 1], and in particular for the suggested value 0.5. In Fig. 13 it is shown a plot of the precision (left) and of the recall (right) for the proposed $\text {FM}_{\text {FG}}$ method, two state-of-the-art face detection methods based on deep convolutional neural networks: the multiscale MTCNN [14] and Retina Face [13], and the well-known Viola Jones [4]. The plots show that the proposed method has a precision superior to all other methods in the IoU range [0, 0.40] and up to IoU$=0.66$ is below only MTCNN. In the interval [0.66, 0.76] the precision of $\text {FM}_{\text {FG}}$, Retina Face and Viola Jones are very close, while precision of MTCNN is quite superior in this range. In the IoU range of [0.76, 1], $\text {FM}_{\text {FG}}$ precision surpasses all the other methods again. Regarding recall, in the right plot in Fig. 13 we see how $\text {FM}_{\text {FG}}$ finds less faces in the FDDB dataset, at least in the range [0, 0.78], with MTCNN and Retina Face showing the highest recall scores.

**Fig. 12:**
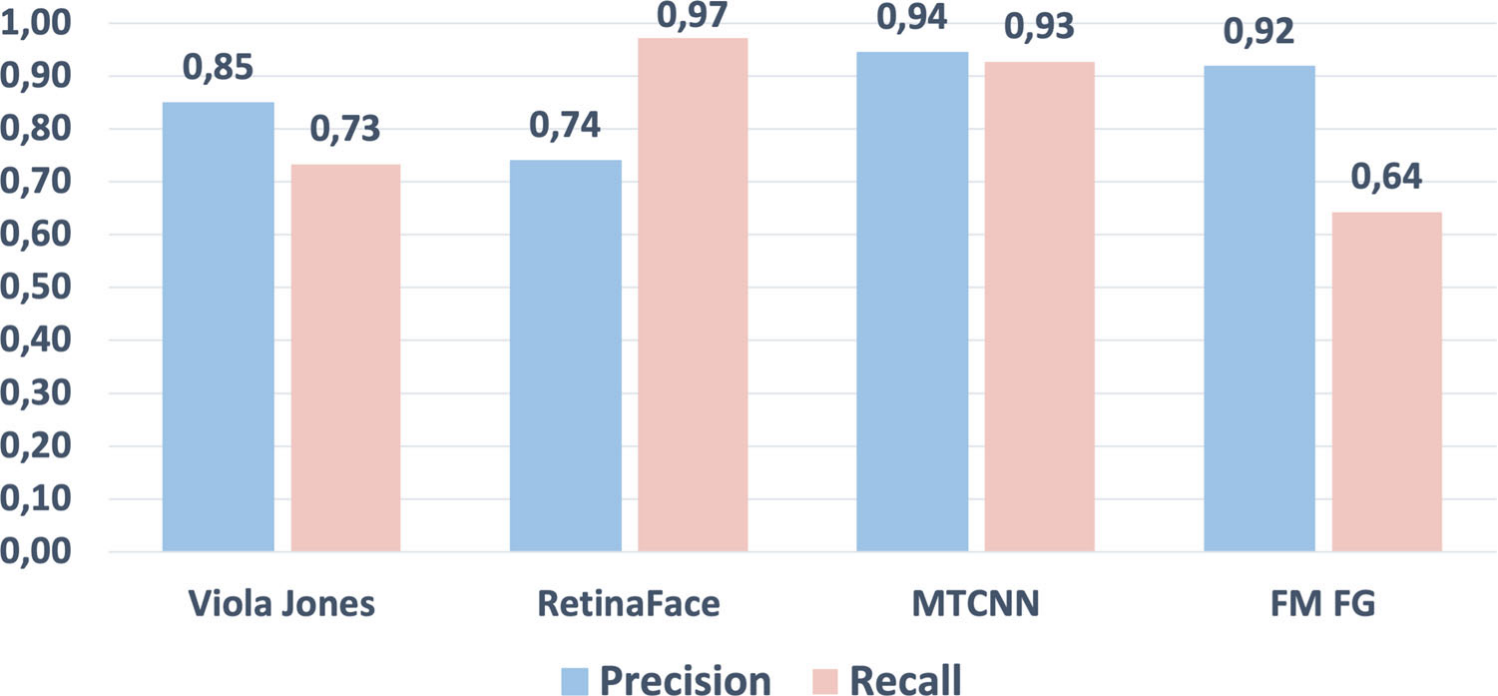
Precision and recall at the $IoU=0.5$ for our proposed method $\textrm{FM}_\textrm{FG}$, and MTCNN, Retina Face and Viola Jones

In Fig. 12, the precision and recall values are reported at the IoU threshold of 0.5. MTCNN has high scores of both precision and recall at this threshold, Retina Face detects even more faces than MTCNN at the expense of a much higher false positive numbers. Our proposed method has the lowest recall, but still reasonable for several face detection applications. It has however a very high precision, a behavior that is also typical of human observers [25].

**Fig. 13:**
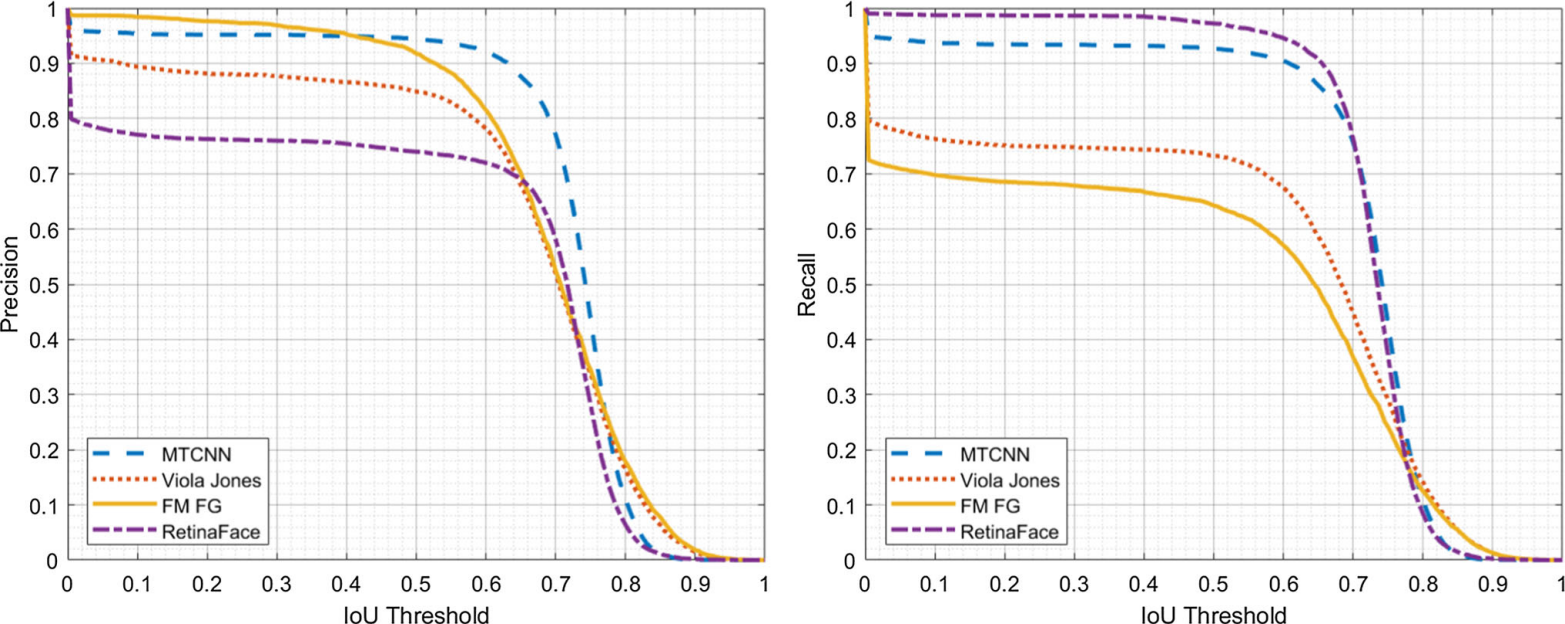
Precision and Recall at varying IoU thresholds for $\textrm{FM}_\textrm{FG}$, MTCNN, Retina Face and Viola Jones

**Fig. 14:**
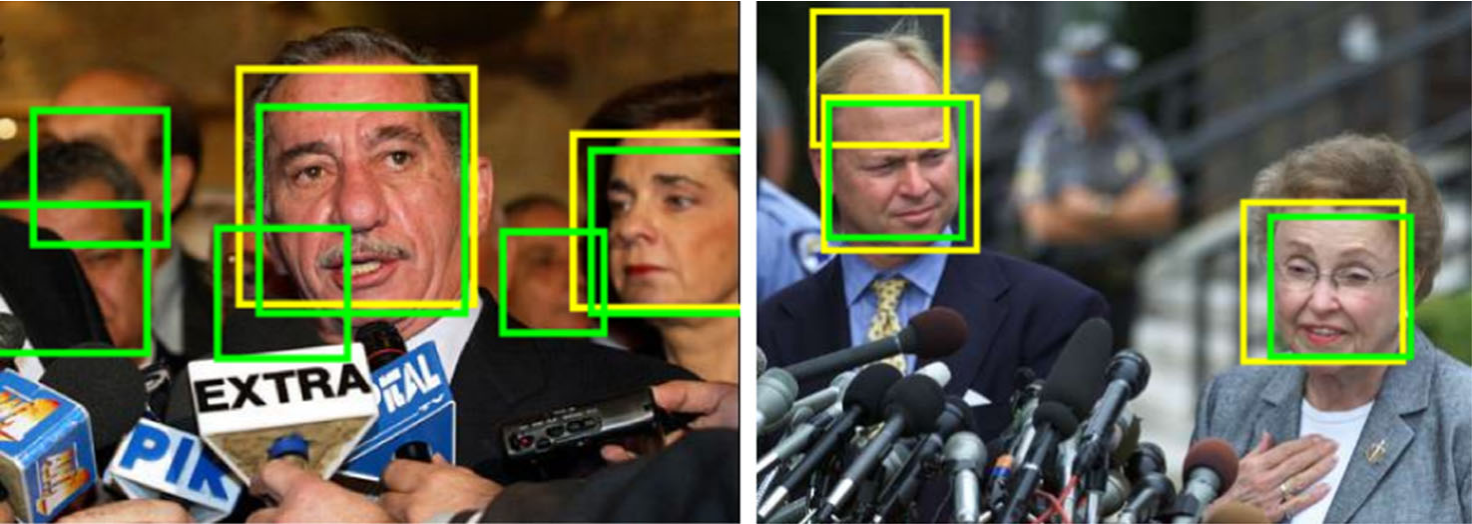
Some examples of system failure of $\text {FM}_{\text {FG}}$ on two images from the FDDB dataset. The detected face are bounded by yellow boxes and the ground truths by green boxes

**Table 2:**
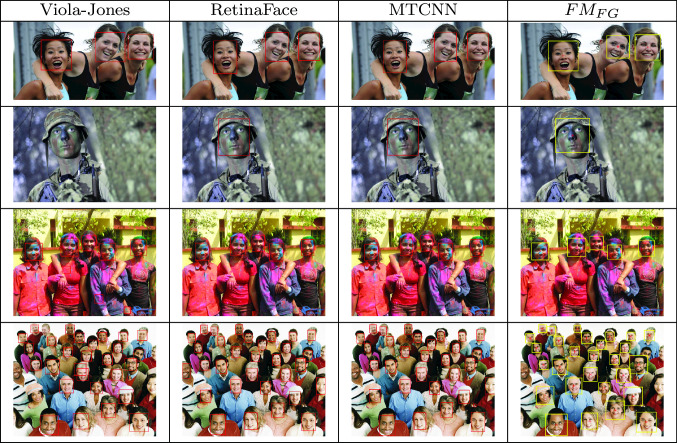
Examples of face detection 4 images of WIDER FACE

From the left, Viola Jones, RetinaFace, MTCNN and $\text {FM}_{\text {FG}}$

In Fig. 14 we can see some instances of false negative and false positive detections of the model $\text {FM}_{\text {FG}}$ (detected faces are in yellow boxes while ground truths are in green boxes). The model clearly struggles with heavily occluded, blurred faces (image on the left). This is explained from the fact that the facial areas our model is based on are not visible at all in these images. On the right image in figure we can see a (rare) case of false positive, which is a “double detection” of a face.

The lower recall of the proposed $\text {FM}_{\text {FG}}$ method with respect to the state of the art was somehow expected, as the model was trained on the aligned partition of LFW which contains only slight rotations away from the frontal pose. Moreover, the binary classifier limits the detection of possible face regions. However, while the model is not able to detect all faces, the number of false positive is very low, better than all other methods and comparable to MTCNN, so it could be exploited in contexts where low false positives are desirable.

In Table 2, we compare $\text {FM}_\textrm{FG}$ to the other state-of-the-art methods on 4 selected challenging images from the Face Detection Benchmark WIDER FACE [39] containing expressions, makeup and occlusions. Viola–Jones shows a high number of false positive (4 on the third image from the top) and cannot detect the soldier in the second image from the top. Our method does not detect any false positives and is comparable to MTCNN and RetinaFace for not occluded faces.

## Conclusions

Multi-scale face detectors could represent a valid, lighter and explainable alternative to deep learning models for face detection. In this work, we propose to use human fixations to guide the construction of a bottom-up multi-scale model, by selecting the scales at which feature clusters are closest to human fixations clusters. Extensive experiments on the FDDB dataset show that the model based on the scales that best correlate with human attention achieves a good face detection performance, with the best recall with respect to any of the models based on different scales (and different number of scales) paired with a very high precision. The model construction is versatile and could be realized for other object classes of interest, such as cars, pedestrian, etc. In this regard, it would be intriguing to investigate whether human attention on these classes can enhance the model’s performance in a similar manner as it does for faces.

In terms of performance, there are multiple ways to improve recall rates. A first approach could be to explore alternative descriptors besides HOGs, enabling the utilization of the model’s coarser scale $\sigma _{10}$ that strongly aligns with human attention. The multi-scale model could also be developed into a full DPM model by using the probability density maps information to define slight movements of the different patches which will likely improve the recall. Finally, by adopting a non-binary SVM classifier that enables the choice of a threshold, it would be possible to optimize the precision versus recall trade-off according to the face detection context.
